# Cost-effectiveness of i-Sleep, a guided online CBT intervention, for patients with insomnia in general practice: protocol of a pragmatic randomized controlled trial

**DOI:** 10.1186/s12888-016-0783-z

**Published:** 2016-04-02

**Authors:** Tanja van der Zweerde, Jaap Lancee, Pauline Slottje, Judith Bosmans, Eus Van Someren, Charles Reynolds, Pim Cuijpers, Annemieke van Straten

**Affiliations:** Department of Clinical, Neuro, and Developmental Psychology, Section Clinical Psychology, Faculty of Behavioural and Movement Sciences, Vrije Universiteit Amsterdam, Amsterdam, The Netherlands; EMGO institute for Health Care and Research, VU University Medical Centre, van der Boechorststraat 7, 1081 BT Amsterdam, The Netherlands; Department of Clinical Psychology, Faculty of Social and Behavioural Sciences, University of Amsterdam, Amsterdam, The Netherlands; Academic Network of Family Medicine (ANH), VU University Medical Center, van der Boechorststraat 7, 1081 BT Amsterdam, The Netherlands; Department of Health Sciences, Faculty of Earth and Life Sciences, Vrije Universiteit Amsterdam, Amsterdam, The Netherlands; Department of Sleep and Cognition, Netherlands Institute for Neuroscience, an Institute of the Royal Netherlands Academy of Arts and Sciences, Amsterdam, The Netherlands; Departments of Integrative Neurophysiology and Psychiatry, Centre for Neurogenomics and Cognitive Research, VU University Medical Centre, Amsterdam, The Netherlands; Department of Psychiatry, University of Pittsburgh, Pittsburgh, PA USA

**Keywords:** CBT-I, Cognitive behavioural therapy, Cost-effectiveness, General practice, Insomnia, Online treatment, Pragmatic randomized controlled trial

## Abstract

**Background:**

Insomnia is a highly prevalent disorder causing clinically significant distress and impairment. Furthermore, insomnia is associated with high societal and individual costs. Although cognitive behavioural treatment for insomnia (CBT-I) is the preferred treatment, it is not used often. Offering CBT-I in an online format may increase access. Many studies have shown that online CBT for insomnia is effective. However, these studies have all been performed in general population samples recruited through media. This protocol article presents the design of a study aimed at establishing feasibility, effectiveness and cost-effectiveness of a guided online intervention (i-Sleep) for patients suffering from insomnia that seek help from their general practitioner as compared to care-as-usual.

**Methods/design:**

In a pragmatic randomized controlled trial, adult patients with insomnia disorder recruited through general practices are randomized to a 5-session guided online treatment, which is called “i-Sleep”, or to care-as-usual. Patients in the care-as-usual condition will be offered i-Sleep 6 months after inclusion. An ancillary clinician, known as the psychological well-being practitioner who works in the GP practice (PWP; in Dutch: POH-GGZ), will offer online support after every session. Our aim is to recruit one hundred and sixty patients. Questionnaires, a sleep diary and wrist actigraphy will be administered at baseline, post intervention (at 8 weeks), and at 6 months and 12 months follow-up. Effectiveness will be established using insomnia severity as the main outcome. Cost-effectiveness and cost-utility (using costs per quality adjusted life year (QALY) as outcome) will be conducted from a societal perspective. Secondary measures are: sleep diary, daytime consequences, fatigue, work and social adjustment, anxiety, alcohol use, depression and quality of life.

**Discussion:**

The results of this trial will help establish whether online CBT-I is (cost-) effective and feasible in general practice as compared to care-as-usual. If it is, then quality of care might be increased because implementation of i-Sleep makes it easier to adhere to insomnia guidelines. Strengths and limitations are discussed.

**Trial registration:**

Netherlands Trial register NTR 5202 (registered April 17^st^ 2015).

## Background

Insomnia is a highly prevalent disorder, characterized by difficulty initiating and/or maintaining sleep for at least three nights per week for at least three months, causing clinically significant distress or impairment in daily functioning [1]. One third of the general population shows symptoms of the disorder and about 10 % of the general population meets all criteria of insomnia disorder [2]. The disorder often persists for years [3]. The economic burden of insomnia is considerable; poor sleepers cost around ten times more than good sleepers due to increased healthcare use, work absenteeism and lower work productivity [4, 5]. Insomnia is a significant problem in itself, but it is also associated with the development of other problems. Patients suffering from insomnia are at greater risk of developing comorbid mental health problems such as depression and anxiety [6–9] and of developing cardiovascular problems, contributing to an increased mortality risk [10, 11].

The high prevalence and burden of insomnia calls for (cost-) effective treatment. The most common treatment for insomnia is medication; around 60 % of patients seeing their general practitioner (GP) with complaints of insomnia receive a prescription for benzodiazepines or benzodiazepine-related medication [12, 13]. Meta-analyses have demonstrated that benzodiazepines are effective in enhancing sleep in the short run, but have negative side effects, e.g. headaches, drowsiness, dizziness, dependence [14, 15]. Furthermore, there is limited evidence for the effects of sleep medication used in the long run [16]. There is also little evidence that improvement of sleep persists when sleep medication is withdrawn [17].

Various non-pharmacological alternatives for the treatment of insomnia are available. These treatments can be classified as educational (psycho-education, sleep hygiene), behavioral (relaxation, sleep restriction, stimulus control) or cognitive (paradoxical intention, identifying and challenging dysfunctional thoughts about sleep) [18–23]. Since the 1990s it has become popular to offer those treatments in (various) combinations, referred to as Cognitive Behavioral Therapy for Insomnia (CBT-I). Several reviews have concluded that CBT-I is effective and has longer lasting effects than medication. Even though some side effects might occur in CBT-I, mainly due to more pronounced fatigue because of sleep restriction, those side effects usually pass quickly [24]. Therefore, CBT-I is considered the preferred treatment for insomnia [17, 20, 25–30] and is recommended in several insomnia guidelines (e.g. NICE guidelines [31] and Dutch guidelines [32]). Practice shows that there is a gap between what is advocated by the guidelines (i.e.: CBT-I) and what is offered in practice: despite considerable evidence for the effectiveness of face-to-face CBT-I in general practices [33], GPs tend to prescribe medication. A possible reason is that GPs generally have insufficient time or knowledge to provide the CBT-I themselves [33]. Furthermore, there are not enough qualified psychologists available to offer CBT-I. Online CBT-I may provide a possible solution and enable GPs to offer patients accessible evidence-based treatment. During the past decade, e-health has been introduced steadily into mental healthcare. Many e-health programs have been developed for different disorders [34–36]. Meta-analyses have shown that these (guided) self-help programs, defined largely as standardized therapies that patients work through independently and at their own convenience [37], are effective for a variety of mental health disorders [38]. Meta-analyses on trials on self-help for insomnia [39–42] have shown medium to large effects. A recent meta-analysis showed that effect sizes for online CBT-I were comparable to those of CBT-I delivered face-to-face [43].

In general, e-health treatments are more effective when delivered with some form of guidance or coaching [40, 44, 45]. This guidance can be delivered by non-specialists such as psychology students, nurses or psychological wellbeing practitioners (PWP; in Dutch: POH-GGZ). In the Netherlands almost every GP practice employs a PWP, usually a psychiatric nurse. This offers an excellent opportunity to implement online treatment for insomnia supported by PWPs in the general practice.

The treatment to be investigated in the proposed study, i-Sleep, proved to be feasible, acceptable and effective [42]. However, in this previous study the participants were recruited through a popular website offering information on insomnia to the general public (www.insomnie.nl). This may have resulted in a study population that is, through self-selection, significantly different (e.g., more highly motivated, more technology-savvy) than patients turning to their GP for help. In the proposed study, patients will be recruited via GP referral. This means that we target a population of patients living with insomnia disorder who are actively seeking help, i.e. a representation of general practice. We will examine the (cost-) effectiveness of online guided CBT-I (i-Sleep) from a societal perspective in a direct comparison with care-as-usual.

### Aims and hypotheses

At the moment G.P. care for insomnia is mainly consisting of sleep hygiene and prescribing medication and, therefore, is often suboptimal, i.e. not adhering to guidelines. The objective of this pragmatic trial is to evaluate the effectiveness and cost-effectiveness of an online CBT-I intervention, i-Sleep, for patients with insomnia in comparison with care-as-usual. Patients will receive online coaching by PWPs working in general practices. We expect the intervention to improve the quality of care for insomnia (i.e., lead to higher improvement rates because more people receive care according to the guideline) and to be cost-effective compared to care-as-usual because of low intervention costs, less healthcare utilization and less lost productivity. We expect that patients in the intervention group will show a reduction in insomnia complaints and improved sleep parameters, suffer fewer daytime consequences, and have lower costs related to healthcare utilization and lost productivity than patients in the care-as-usual group.

## Methods/design

### Study design

This study is a pragmatic randomized controlled trial with an economic evaluation from a societal perspective alongside. Patients referring to their GP with insomnia are randomized to either the online intervention group, who will gain access to the guided online intervention, or the control group, who will receive G.P. care-as-usual and will be offered the investigated guided online intervention six months after inclusion. The study protocol, information brochure, questionnaires, recruitment material and informed consent form were approved by the Medical Ethics Committee of the VU University Medical Centre (METC VUmc, registration number 2015/258). The trial is registered with the Netherlands Trial Register (NTR 5202).

### Inclusion and exclusion criteria

Patients aged 18 and over who turn to their GP for help with insomnia complaints are evaluated for eligibility. Inclusion criteria are assessed using a screening questionnaire (t0) and include: difficulty initiating or maintaining sleep for at least 30 minutes a night, for at least three nights a week, for at least three months, causing clinically significant distress and/or impairment in daily functioning, i.e. meeting criteria for a DSM-5 diagnosis of insomnia [1]. Exclusion criteria are: no access to the internet, inadequate proficiency in Dutch, meeting criteria of the diagnosis of sleep apnea (assessed by the GP and in the screening questionnaire using the Screening for Sleeping disorders [46], pregnancy or breast feeding during the trial, working in night shifts, psychological treatment in the last six months, being suicidal (assessed using 5 items from the MINI diagnostic interview [47]), and schizophrenia or psychotic disorder (“Have you been diagnosed with schizophrenia or psychosis?”, to which participants reply ‘Yes’ or ‘No’). Other comorbid psychological disorders and somatic diseases are allowed. The use of (sleep) medication is also allowed and tracked.

### Recruitment

Patients are recruited through general practices. Recruitment leaflets are distributed in waiting rooms, and GPs are instructed to alert insomnia patients to the trial. Patients interested in participating are directed to a website for more information and to leave their contact details. Depending on speed of inclusion, we will also ask GPs to contact patients that presented with insomnia in the 12 months before the start of the trial (database search).

**Fig. 1:**
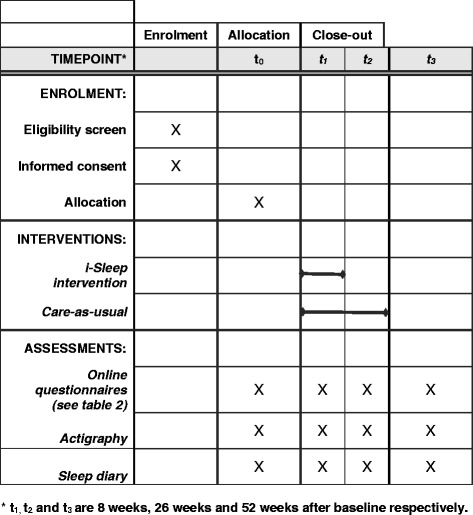
Schedule of enrolment, interventions, and assessments [75]

Patients that enrol receive an email containing background information and a link to the screening questionnaire. If eligible, patients are sent an information kit containing a study brochure, sleep diary, actigraphic watch, informed consent form, and stamped return envelope to confirm participation. Patients complete the baseline measurement by filling in a sleep diary and wearing an actigraphic watch for a week. For a detailed representation of participant flow in this trial, see Fig. 1.

Figure 1. Flowchart of the study design.

### Randomization

After inclusion patients are randomized by an independent researcher using random sequence block randomization (blocks of 2, 4 or 6), stratified by whether or not they have used sleep medication in the past 4 weeks and by individual PWP, on a 1:1 ratio. After inclusion of a patient the independent researcher reveals the next randomization outcome. Due to the nature of the study, blinding of the researchers, PWPs or patients is not possible.

### The intervention: guided online intervention i-Sleep for insomnia

The online intervention i-Sleep, developed at the Vrije Universiteit Amsterdam (AS, JL, TZ), is based on a mixture of self-help materials, research literature and interviews with patients. The treatment consists of online lessons and includes the elements that are commonly incorporated in face-to-face CBT-I [19–23]. Initially the treatment consisted of 6 lessons. Based on the first trial on i-Sleep [42] we decided to combine the first two lessons (on psycho-education and sleep hygiene) into one because most patients were already familiar with (part of) this information. The topics during the five lessons are explained in Table 1. The intervention is offered with online guidance from a coach. The aim of the feedback is to comment on exercises, clarify information and motivate the patient to persist in carrying out the course and the requested behavioral changes [44]. The coach responds to every session the patient completes. We advise patients to do one lesson every week but they can take some more time if needed. However, during this study, coaching will stop 8 weeks after patients received login details even in case patients do not finish the entire treatment.

**Table 1:** The session of online treatment i-Sleep

Session	Content
1	Psycho-education, and sleep hygiene.
2	Stimulus control and sleep restriction: patients are taught to use the bedroom only to sleep and to get in and out of bed at the same time every day. Furthermore they are asked to restrict this the time in bed to the average amount of night-time sleep. The initial sleep window prescribed is based on sleep diary parameters over 7 nights, with a minimum of a 5 hour sleep window. When sleep efficiency (percentage of time spent in bed that the person is asleep) is below 85 %, time in bed is restricted. Above 85 %, the sleep window can be lengthened. Position of the sleep window is as patients prefer (going to bed later or getting up earlier). Daytime napping is discouraged. If unavoidable, no longer than 30 minutes and not after dinner. Sleep restriction will be continued in session 3, 4 and 5 according to need.
3	Relaxation exercises and exercises to minimize worrying.
4	Dysfunctional cognitions about sleep are addressed: the basics of cognitive therapy are explained and the most common erroneous ideas about insomnia are discussed.
5	Summary and plan for the future.

Patients in the intervention condition will receive guidance from a psychological wellbeing practitioner (PWP) in each general practice, usually a (psychiatric) nurse. We will provide training for the PWP in CBT-I as well as in providing online feedback. The training will consists of a 1.5 hour workshop, guided practice with the online course and feedback training. PWPs will be supervised by the first author (TZ) and an experienced CBT psychologist. Patients in both conditions are also allowed to receive care-as-usual from their GP or the PWP.

### Care-as-usual group

We will provide the participating GPs in our study with a written version of the Dutch insomnia guidelines [32]. The core message of the guideline is formulated as: (1) the preferred insomnia treatment is non-pharmacological, (2) the GP might consider medication in exceptional situations, and only short term, (3) the preferred treatment for short-term sleep problems is psycho-education and information about sleep hygiene, (4) for longer term sleep problems the preferred treatment is a combination of stimulus-control, sleep restriction, relaxation and structured exercise [32]. We will not interfere with the care that the GPs offer their patients. After online data collection, we will extract information on consumption of GP-care and prescription of medication from GP electronic records. Also, we will inquire from patients themselves how many nights was medication used in the last week using the sleep diary.

### Assessments

Four assessments will take place: at baseline (T0), 8 weeks post-treatment (T1), 6- (T2) and 12-month (T3) follow-up. For an overview of the measurements see Table 2. Questionnaires will be administered online. Additionally, participants will receive an actigraphic watch and a sleep diary by mail at all assessments. Patients are asked to wear the actigraphic watch for 7 days while also filling out the sleep diary for these same days [48]. Additionally, data extracted from the electronic medical records in general practice will be used to assess consumption of care and prescription of sleep medication, up to 12 months follow-up.

**Table 2:** Measurements and instruments at different point of assessment

Measurement	Instrument	Baseline (T0)	T1, T2 and T3 (at 8, 26 and 52 weeks)
Background characteristics	n/a	X	-
History of insomnia	n/a	X	
Alcohol use	AUDIT	X	X
Insomnia severity	ISI	X	X
Anxiety and depression	HADS	X	X
Daytime insomnia consequences	Espie et al., 2012	X	X
Daytime functioning	WSAS	X	X
Fatigue	FSS	X	X
Quality of life	EQ-5D-5 L	X	X
Healthcare utilization & work absenteeism	TiC-P	X	X
Treatment satisfaction	n/a	-	T1
Actigraphy	Actigraph GT9X Link	X	X
Sleep diary	Consensus diary	X	X
Sleep medication	n/a	X	X

AUDIT: Alcohol Use Disorders Identification Test; ISI: Insomnia Severity Index; HADS: Hamilton Anxiety and Depression Scale; WSAS: Work and Social Adjustment scale; FSS: Fatigue Severity Scale; EQ-5D-L: EuroQoL; TiC-P: Trimbos and iMTA questionnaire on Costs associated with Psychiatric Illness

### Primary outcome: insomnia severity

The primary outcome of the study will be insomnia severity as measured by the Insomnia Severity Index [49]. The ISI consists of 7 items scored on a 5-point Likert scale (0 = not at all, 4 = extremely) and scores range from 0–28, higher scores indicating more severe insomnia. The ISI assesses the nature, severity and the impact of insomnia. Previous research has indicated that the Insomnia Severity Index (ISI) is a valid and reliable instrument to quantify perceived insomnia severity and presents a clinically useful tool in screening as well as outcome measurement. It possesses adequate internal consistency (Cronbach’s alpha = .90), is sensitive to changes in perceived sleep difficulties over time [50] and has been validated for online use [51]. We use a community sample cut-off score of ≥10 to define clinical insomnia [50].

### Secondary outcomes

#### Questionnaires

Daytime consequences suffered due to insomnia will be measured using 6 items on the different domains mentioned in the DSM-5 diagnosis of insomnia disorder: energy, relationships, mood, concentration, productivity, and sleepiness [52] rated on a 5-points scale at each assessment phase (0 = not at all affected through to 4 = very much affected).

Daytime functioning will also be assessed using the 5-item Work and Social Adjustment Scale [53]. Each of the five items measure a domain of functioning: work, home management, social leisure activities (i.e. with others), private leisure activities (i.e. individually) and ability to form and maintain close relationships with others. For each domain of functioning the score ranges from 1 through to 8, with higher scores indicating more severe disruption. The internal consistency of the WSAS ranges from Cronbach’s alpha 0.70 to 0.94 [53].

We will use the Fatigue Severity Scale [54], which consists of 9 items, to measure fatigue. Each item is scored on a 7-point Likert scale, ranging from 1 (strongly disagree) through to 7 (strongly agree). Higher scores indicate more severe fatigue. This scale has shown an excellent internal consistency and reliability (Cronbach’s alpha of 0.93; [55]).

The Hospital Anxiety and Depression Scale consists of 14 items which are scored on a 4-point Likert scale each. Seven items can be summed into a depression a score and the other half into an anxiety score. Higher scores represent more severe anxiety or depression [56]. The HADS is a reliable and valid instrument also in Dutch populations (Cronbach’s alpha of .88, [57]).

The Alcohol Use Disorders Identification Test questionnaire (AUDIT [58]), designed by the World Health Organisation to screen for excessive alcohol consumption, will be used to assess alcohol use, using a 5-point Likert scale (higher scores indicating more alcohol use and related issues). This instrument has a clear cut-off value, is often used and well validated [58]. The total score represents consumption level, dependence and present alcohol-related harm. We will use the standard cut-off of 8 to identify (but not exclude) heavy drinkers which has shown to have good sensitivity and specificity in detecting current social and medical problems related to alcohol.

The EuroQol questionnaire (EQ-5D-5 L) [59] will be used to measure health-related quality of life. Health states will be converted to utility scores using the Dutch EQ-5D-5 L tariff. Quality-adjusted life-years (QALYs) will be calculated by multiplying the utility score of a health state by the time spent in that health state. Transitions between health states will be linearly interpolated.

#### Diary and actigraphy

We will measure sleep estimates derived from both the consensus sleep diary and the use of wrist actigraphy. The sleep estimates are: Sleep Onset Latency (number of minutes it takes to fall asleep after going to bed), Sleep Efficiency (percentage of the total amount of time spent in bed that is spent asleep), Number of Awakenings, Wake After Sleep Onset (total minutes awake after sleep onset), Total Sleep Time and Sleep Quality (a number between 1 and 5 for each night). We will use the Dutch translation of the consensus sleep diary [60] with some minor text adaptations, achieved by consensus among a group of Dutch sleep researchers.

In addition to the subjective sleep evaluations gathered using the questionnaires and sleep diary, objective sleep data will be collected using actigraphy. Actigraphic watches, accelerometers recording motion over short epochs, are non-invasive small devices worn on the non-dominant wrist 24 hours a day. We will equip participants with an Actigraph GT9X Link provided by ProCare, under Actigraph Actilife 6 SmEnterprise licenses. The Actilife software (http://www.actigraphcorp.com/solutions-and-products/software/actilife/) will be used to extract the data from the actigraphic watches. We will use a validated script to calculate different sleep estimates: sleep period, total sleep time, clock time falling asleep, wake up time, percent sleep, wake after sleep onset, number of wake bouts, fragmentation and latency [48, 61]. We will compare the objective and subjective data.

### Cost measures

Healthcare utilization will be assessed in two ways. First we will use data derived from GP records. We will assess the number of consultations with GPs, and general well-being practitioners as well as medication prescriptions. Next, we will ask the patients themselves which other healthcare services they used (GP and other) and which sleep and other medication they took. For this we will use the TiC-P [62] (Hakkaart-van Roijen et al., 2002). The Short Form Health and Labour Questionnaire which is also part of the TiC-P will be used to measure absenteeism from paid and unpaid work, and presentism [62]. Healthcare utilization will be valued using Dutch standard tariffs [63]. Costs associated with lost productivity will be estimated according to the friction cost approach [64].

### Adverse effects

We will investigate potential adverse effects. Adverse effects are defined as (1) worsening of sleep symptoms, and (2) accidents. Adverse effects could be a result of the insomnia disorder itself, but also potentially of the sleep restriction exercise in i-Sleep. We will assess the primary outcome measure in order to identify clinically significant deterioration, e.g. exacerbation of insomnia severity from pre- to post-treatment (ISI change score of 7 points or more [50]. Additionally, we will ask participants at post-test and follow-up 1 and 2 whether they experienced a) falling incidents, b) traffic accidents, c) any other negative events that they feel are related to fatigue/sleepiness. Participants unavailable for post-test and/or follow-up due to dropout will be asked these questions via e-mail. The occurrence of adverse effects in the intervention and in the care-as-usual condition will be compared.

### Sample size

In a previous RCT, the i-Sleep intervention demonstrated an effect size of 1.06 on Sleep Quality (Pittsburgh Sleep Quality Inventory, [42]). However, a recent meta-analysis [40] on self-help for insomnia demonstrates somewhat more modest effect sizes; a Hedges’ *g* of 0.80, 0.66 and 0.55 respectively for sleep efficiency, sleep onset latency and wake after sleep onset at immediate post treatment. For the current study we will use a conservative estimation of a post treatment effect size, a Cohen’s *d* of 0.50, on the primary outcome insomnia severity. Using a power of 80 %, an alpha of 0.05, and a study dropout rate of 20 %, we need to include 80 patients in both conditions, 160 in total, to answer our primary research question.

### Statistical analysis

The study will be conducted in adherence to the Consolidated Standards of Reporting Trials (CONSORT; [65]) statement. For all analyses statistical significance will be set at *p* < .05. In accordance with the intention-to-treat principle, we will use all data independent of treatment or study completion. Missing values will be imputed using multiple imputation for calculation of Cohen’s *d* [66, 67].

Between group interaction effects (time × group) will be tested with Generalized Estimating Equations (GEE). Variables that are not distributed equally or that predict dropout will be added to the model as covariates. This technique is suitable for the analysis of the longitudinal relationship between a continuous outcome variable and several time-dependent and time independent covariates [68].

We will describe the magnitude of the effect (effect size) with Cohen’s *d*. We will calculate this by dividing the difference in scores of the two groups at post-test by the pooled standard deviation of change scores (Cohen’s *d*). This can thus be interpreted as the number of standard deviations the intervention group scores better than the control group [69].

Next, we will compare the intervention and control group with regard to the percentage of patients who have (1) improved and (2) recovered. An ISI score of 10 or lower will be considered recovery. Improvement will be defined as an ISI change score of 7 points or more [50]. This information will be used to calculate the relative risk and the number needed to treat [70].

### Cost-effectiveness and cost-utility analysis

Both cost-effectiveness and cost-utility analyses will be performed. Cost-effectiveness analysis entails measuring the value of the new therapy i-Sleep by calculating the difference in costs between i-Sleep and care-as-usual, and dividing this by the difference in effectiveness of both treatment options (i.e., calculating the cost-effectiveness ratio) [71, 72].

The cost-utility analysis represents the costs associated with gaining one quality-adjusted life year (QALY). Missing cost and effect data will be imputed using multiple imputation. Seemingly unrelated regression will be used to estimate cost and effect differences while adjusting for potential confounders. We will use bootstrapping to estimate uncertainty surrounding the cost-effectiveness estimates to account for the skewed distribution of costs. Cost-effectiveness planes and cost-effectiveness acceptability curves will be estimated to show statistical uncertainty surrounding the cost-effectiveness estimates.

### Budget impact analysis (BIA)

A budget impact analysis (BIA) is used to calculate expected changes in the expenses if the new intervention i-Sleep is adapted into the healthcare system. For the BIA the population of people with insomnia will be estimated using Dutch epidemiological data. We will extrapolate the effectiveness of the treatments using a Markov model based on the estimates obtained from the proposed study. We will evaluate different implementation scenarios (0 %, 50 %, 100 %, subgroups only). Aggregated and disaggregated (e.g. GP/PWP care, secondary care, and productivity losses) total costs per year will be presented for the different perspectives and scenarios. We expect that the intervention will lead to increased work productivity in comparison to care-as-usual, and that the largest economic benefits generated by the intervention are related to reduced productivity losses. Costs associated with the treatment condition are costs of the use of the intervention platform, and invested time of the PWPs.

## Discussion

Insomnia is associated with high personal and societal costs, while the first treatment of choice (CBT-I) is often unavailable. Guided online CBT-I might be a solution. The efficacy of online CBT-I has already been established but an important limitation of those studies is that they are mainly performed in general population samples [73], in which patients might be more motivated or more familiar with the internet than the average insomnia patient who seeks help in the GP practice. To our knowledge, this is the first study performed in general practice thus allowing for generalizability to routine care. Our first aim is to study the effects on insomnia severity and sleep parameters, but also on fatigue, mental health and daytime consequences. Moreover we will compare the cost-effectiveness from a societal perspective between the guided online intervention and care-as-usual in GP practice.

### Strengths and weaknesses

The five major strengths of this study are the fact that a) it is a trial conducted in general practice, where most people with insomnia first turn for help, and where care can be improved; b) we will use objective sleep data through actigraphic watches as well as subjective data through sleep diaries; c) we use an array of outcome measures not only examining sleep itself but also the consequences on mood, anxiety, fatigue and day-time functioning; d) we will examine possible negative effects due to the online intervention and e) we are assessing not only effectiveness but also cost-effectiveness, in a real-life setting of high external validity. The sample will likely be of higher heterogeneity than is the case in studies that recruit participants through a website and will tell us whether this form of treatment is feasible in routine care.

Conducting the trial in general practice ensures that our conclusions will be generalizable to the average patient consulting their GP with sleep problems. Also, guidance can be provided by a psychiatric nurse or PWP. This could (partly) solve the current issue of GPs lacking time and knowledge to deliver CBT-I and the limited availability of trained psychologists. At the same time, the study provides a true test of whether our intervention i-Sleep can be implemented in general practice and therefore be disseminated to a much wider array of patients. We expect to find that i-Sleep can be implemented and provide an alternative to care-as-usual that is more effective in the long term, that is cost-effective, comes with fewer problems such as side-effects and dependence compared to medication, and is well accepted by both the patient and healthcare providers (GP and PWP).

There are also potential weaknesses in our study. First of all, it might be a weakness that our intervention lacks any face-to-face contact. We might also have chosen to offer the treatment in blended format where patients receive a combination of online and face-to-face therapy. Blended care might be more acceptable to the patient as well as the professionals and it has been suggested to be effective in other disorders, e.g. depression [74]. However, for this trial we chose not to blend care, because a purely online treatment was deemed more feasible, more cost-effective and easier to implement. A second potential weakness is that we include a convenience sample of GPs. The participating GPs might be more interested in insomnia than those who do not participate. This means that care-as-usual might be of higher quality than provided on average. Also, the participating GPs offer access to a mostly urban population. A third potential weakness is that we include professionals (PWPs) who are currently employed by GPs but who have varying levels of expertise in insomnia and online treatments. Even though we will train the PWPs, and we will supervise them during the trial, it may lead to heterogeneity in the way treatments are delivered. In other words, the high external validity may lead to less internal validity.

Another potential limitation is the fact that we do not establish the DSM diagnosis based on clinical interviews. Patients are referred to us by the GP who establishes clinically relevant sleep problems. The sleep diary, actigraphy and ISI provide us with further information about the severity of the insomnia. Using online questionnaires eliminates the risk of interviewer bias and it ensures relatively easy follow-up measurements while minimizing the burden for participants.

Also, it may be a disadvantage that participants do not gain access to the online treatment immediately: the trial involves a series of practical steps and registration that take some time between enrollment and actual participation. This may influence patients’ motivation negatively.

In summary, online CBT for insomnia might be a cost-effective alternative to care-as-usual, and the proposed study aims to establish that online guided intervention such as our i-Sleep module can be successfully used in primary care. The availability of a web-based intervention could be an innovative way for GPs to adhere to the insomnia guidelines and offer patients optimal care while efficiently using limited resources.

### Trial status

The first participants are being recruited.

### Availability of Data and Materials

Collected data will be made accessible (without compromising anonymity) at the end of the data collection phase.

## Abbreviations

AUDIT: Alcohol Use Disorders Identification Test
BIA: Budget Impact Analysis
CAU: Care-as-usual
CBT: Cognitive Behavioral Treatment
CBT-I: Cognitive Behavioral Treatment for Insomnia
CONSORT: Consolidated Standards of Reporting Trials
DSM: Diagnostic and Statistical Manual
FSS: Fatigue Severity Scale
GEE: Generalized Estimating Equations
GP: General Practitioner
HADS: Hamilton Anxiety and Depression Scale
ISI: Insomnia Severity Index
NTR: Nederlands Trial Register
POH-GGZ: Praktijkondersteuner Huisarts-Geestelijke Gezondheidszorg
PWP: Psychological Wellbeing Practitioner
QALY: Quality Adjusted Life Year
TiC-P: Trimbos/iMTA questionnaire for Costs associated with Psychiatric Illness
WSAS: Work and Social Adjustment Scale
